# Satisfactory thumb metacarpophalangeal joint stability after ligament reconstruction with flexor digitorum superficialis in children with radial longitudinal deficiency

**DOI:** 10.1177/17531934231187813

**Published:** 2023-07-13

**Authors:** Ida Neergård Sletten, Jarkko Jokihaara, Anne Birgit Stavenes, Mona Irene Winge

**Affiliations:** 1 Division of Orthopaedic Surgery, Oslo University Hospital, Oslo, Norway; 2 Department of Hand Surgery, Tampere University Hospital, Tampere, Finland; 3 Faculty of Medicine and Health Technology, Tampere University, Finland

**Keywords:** Congenital thumb hypoplasia, flexor digitorum superficialis, ligament reconstruction, opponensplasty

## Abstract

We investigated thumb joint stability and patient-reported and functional outcomes a minimum of 1 year after flexor digitorum superficialis opponensplasty and ligament reconstruction in 23 thumbs of 20 consecutive children with radial longitudinal deficiency. In total, 15 thumbs had preoperative multidirectional instability in the metacarpophalangeal joint. We reconstructed 22 ulnar and 16 radial collateral ligaments. At follow-up, all the metacarpophalangeal joints were stable ulnarly. Seven metacarpophalangeal joints were unstable radially despite ligament reconstruction but had no related complaints. We recommend the flexor digitorum superficialis opponensplasty as a safe and reliable procedure in hypoplastic thumbs to create stability and augment thumb strength.

**Level of evidence:** IV

## Introduction

All patients with a congenital radial longitudinal deficiency (RLD) have thumb hypoplasia (Forman et al., 2020), ranging from a slightly short and narrow thumb (type I) to thumb aplasia (type V) (Manske and McCarroll, 1992). Type I thumbs do not need treatment, and the preferred treatment for types IIIb to V is pollicization of the index finger (Carter et al., 2022). There is more variation in the treatment of the most common types II and IIIa (Forman et al., 2020), which are characterized by a tight first web, instability in the thumb metacarpophalangeal joint (MCPJ), and thenar hypo- or aplasia; in type IIIa there are also anomalous extrinsic tendons (Manske and McCarroll, 1992). A surgical release and deepening of the web increases grip span, a stabilization of the MCPJ improves grip and pinch, and an opponensplasty creates or enhances palmar abduction and opposition strength. In a recent international survey of congenital hand surgeons on opponensplasty (Wall et al., 2021), 81% of the responders reported that they preferred using the middle or ring finger flexor digitorum superficialis (FDS) (Kozin and Ezaki, 2010) and 18% the abductor digiti minimi (ADM) (de Roode et al., 2010). Previous studies have reported good outcomes in FDS opponensplasties (Christen and Dautel, 2013; de Kraker et al., 2016; Smith et al., 2012; Vuillermin et al., 2016). However, there are different recommendations for treating multidirectional MCPJ instability. Some report adequate stability after ligament reconstruction with FDS tendon slips (Christen and Dautel, 2013; Vuillermin et al., 2016), whereas others recommend a primary chondrodesis or fusion (de Kraker et al., 2016; Smith et al., 2012; Tonkin, 2014).

We began reconstructing type II and IIIa hypoplastic thumbs at Oslo University Hospital in 2009. We used the FDS-ring for the opponensplasty and MCPJ ligament reconstruction in all our patients. The primary aim of this study was to investigate whether we achieved adequate stability for uni- and multidirectionally unstable MCPJs. The secondary aim was to explore patient-reported and functional outcomes.

## Methods

We conducted the study according to the Helsinki declaration and have reported it according to the Strengthening the Reporting of Observational Studies in Epidemiology (STROBE) statement (Vandenbroucke et al., 2007). Our institutional review board approved the research protocol, and all the children’s caregivers signed a written consent at inclusion. The inclusion criteria were: FDS-ring opponensplasty, including MCPJ ligament reconstruction for a Manske type II or IIIa hypoplastic thumb; a minimum follow-up time of 1 year; and a minimum patient age of 5 years at the study follow-up. We identified 22 eligible participants (25 hands) using an electronic search in our patient records with the International Classification of Diseases 10th Revision code Q71.4 and a manual search in our operation logbooks between 2009 and 2020. A total of 20 patients with 23 reconstructed thumbs (92%) agreed to participate and completed the follow-up assessment between 2020 and 2022.

**Figure 1.**
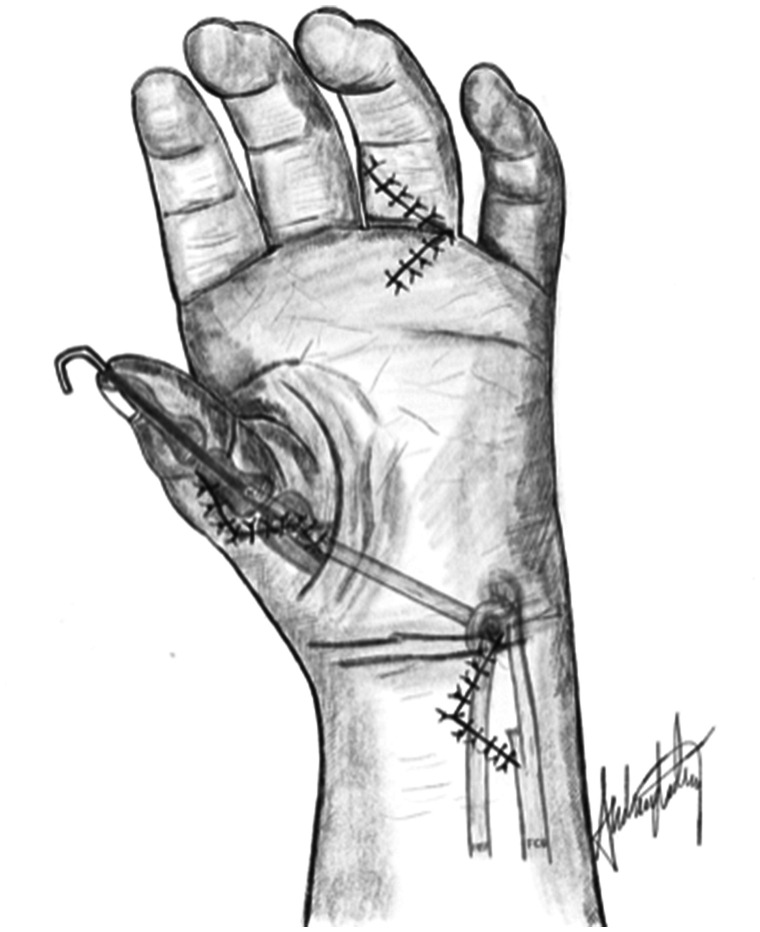
The flexor digitorum superficialis tendon from the ring finger (FDS-ring) is detached distally and pulled proximal to the wrist level. It is secured by a pulley (here: the flexor carpi ulnaris loop) and passed subcutaneously to the thumb. The tendon is inserted into the distal dorsoradial part of the thumb metacarpal with the thumb in maximal palmar abduction with the wrist extended 20°. Illustration: A. Lødrup.

**Figure 2.**
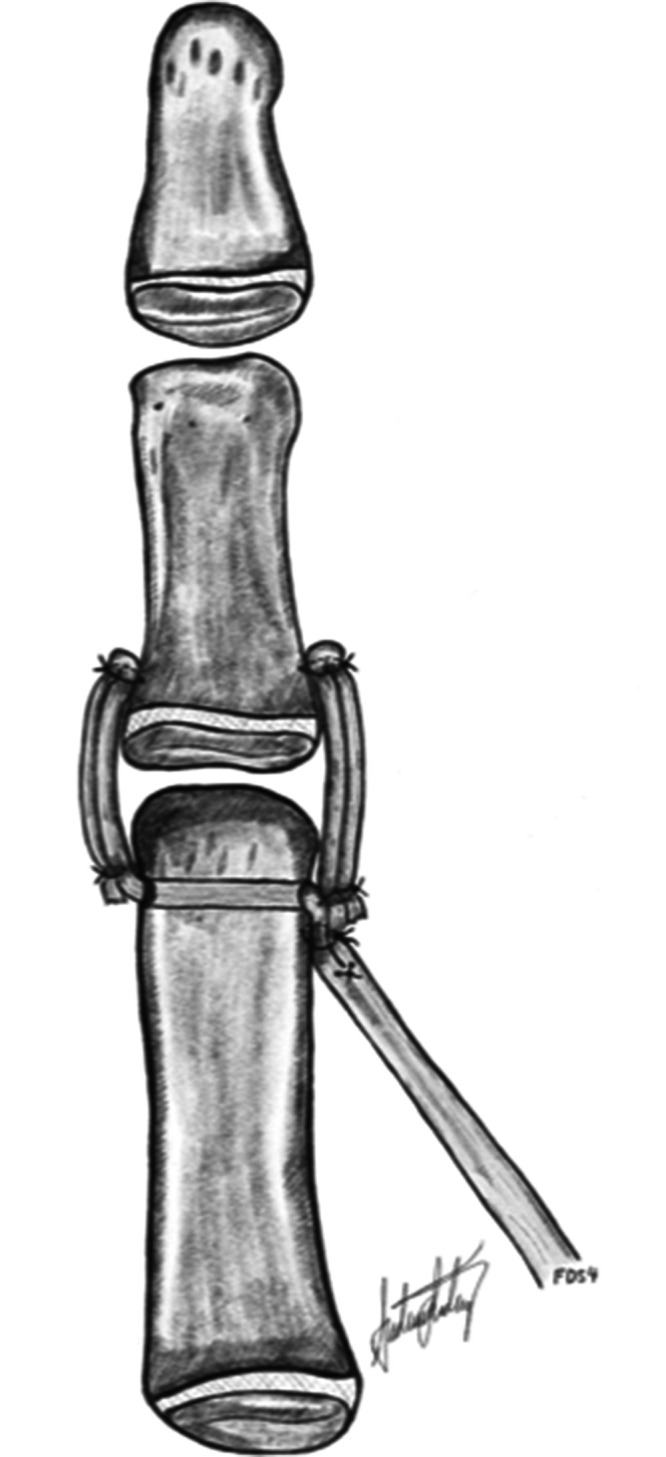
The flexor digitorum superficialis tendon from the ring finger (FDS-ring) is secured with osteosutures into the distal dorsoradial aspect of the thumb metacarpal just proximal to the pre-drilled mid-axial first metacarpal head bone tunnel. The tendon splits are separated, and the ulnar one is passed through the tunnel. Both splits are anchored to periosteal slits in the metaphysis of the proximal phalanx and doubled back over the joint and attached to themselves to reinforce the reconstruction. Illustration: A. Lødrup.

Two surgeons (MW and INS) carried out all except two of the earliest procedures. We constructed a palmar aponeurosis (Christen and Dautel, 2013) or flexor carpi ulnaris (FCU) pulley (Kozin and Ezaki, 2010) for the FDS-ring opponensplasty in all hands (Figure 1) and performed a first web-plasty in all but one hand (Table 1). Before passing the FDS tendon subcutaneously to the thumb, we used a cannulated drill to create a mid-axial bone tunnel in the head of the thumb metacarpal. We tensioned the FDS with the thumb in maximal palmar abduction and the wrist extended 20°, and secured the tendon into the periosteum of the distal dorsoradial area of the thumb metacarpal, just proximal to the bone tunnel (Figure 2). We secured alignment in the MCPJ with a K-wire in 20 thumbs, then reconstructed the MCPJ ligaments with the two terminal slips of the FDS-ring tendon. One slip was passed through the tunnel to the ulnar side (Christen and Dautel, 2013). We first reconstructed the ulnar collateral ligament (UCL) in all 15 thumbs with multidirectional MCPJ instability, followed by the radial collateral ligament (RCL) reconstruction. We attached the tendon slips to ulnar and radial mid-axial proximal phalanx periosteal slits, doubling back over the MCPJ if long enough. In thumbs with additional interphalangeal joint (IPJ) instability, we reconstructed the ligaments by imbricating a local capsular flap. One type IIIa thumb had a preoperative positive dorsal shift in the carpometacarpal joint (CMCJ), which we treated with a Y-V joint-plasty. We divided abnormal intertendinous connections in three thumbs. Postoperatively, we immobilized the hand in a cast for a median of 5.4 weeks (interquartile range [IQR]: 5.0 to 5.7) and removed the K-wires at the time of cast removal. The children wore a protective daytime thumb splint for 2 to 4 weeks and a first web night splint for 3 months.

**Table 1.** Surgical procedures.

	Manske type II (9 thumbs)	Manske type IIIa (14 thumbs)
First web-plasty		
Four-flap plasty	7	7
Simple Z-plasty	2	4
Dorsoradial transposition flap	0	2
None	0	1
FDS-ring opponensplasty		
FCU-pulley	3	2
Palmar aponeurosis pulley	6	12
Ligament reconstruction		
MCPJ UCL	8	14
MCPJ RCL	7	9
IPJ UCL	3	1

FCU: flexor carpi ulnaris; FDS-ring: ring finger flexor digitorum superficialis; IPJ: interphalangeal joint; MCPJ: metacarpophalangeal joint; RCL: radial collateral ligament; UCL: ulnar collateral ligament.

We checked the patient records for intra- and postoperative complications (reoperations, infection, suture granuloma or irritation, healing problems, nerve injury and donor-site morbidity) and included any adverse events reported by the children or their caregivers.

At the study follow-up, a congenital hand surgeon (INS or MW) assessed whether the thumb joints were unstable (yes or no?) or if the first web space was tight (yes or no?). We defined MCPJ and IPJ instability as a lack of a definite endpoint at ligament testing, indicating the need for surgical stabilization. The CMCJ was defined as unstable if we found a positive dorsal shift (Mende et al., 2022). The first web was defined as tight when the surgeon considered that a web-plasty would be beneficial for grasping large objects.

The children performed the Thumb Grasp and Pinch Assessment (T-GAP), and a senior paediatric occupational therapist (OT) (ABS) scored the video clips (Kollitz et al., 2018; Tomhave et al., 2019). There are currently no T-GAP reference values, but we have previously reported its validity and reliability in reconstructed thumbs in a subset of this patient cohort (Sletten et al., 2023).

We used the Pollexograph (de Kraker et al., 2009) to measure active palmar abduction and adduction of the thumb, and the Kapandji score to quantify opposition (Kapandji, 1986). The OT used a goniometer to measure the active range of motion (ROM) in the MCPJ and IPJ, and active radial abduction between the thumb and index metacarpal with the palm of the hand flat on a table. We measured thumb retropulsion from the table to mid-pulp with maximum lift-off of the thumb (Mende et al., 2022), and used the Jamar® Plus+ Digital Hand Dynamometer and Digital Pinch gauge (Patterson Medical Holdings, Inc., Bolingbrook, IL, USA) to measure grip strength, tip, lateral (key) and palmar (tripod) pinch. We have presented strength outcomes as a percentage of age- and sex-adjusted normative values (Bohannon et al., 2017; McQuiddy et al., 2015).

The WIMEC and WIMMECSS scores are functional instruments for assessing hypoplastic thumbs (Mende and Tonkin, 2021). WIMEC (an acronym for W, first web; I, intrinsic function; M, MCPJ stability; E, extrinsic function; C, CMCJ stability) is a score used for recording five anatomical categories; WIMMECSS is an extended version that includes measurements of ROM (M_2_, mobility) and strength (S_1_, pinch; S_2_, grip). We used our measurements of radial abduction, Kapandji score, MCPJ stability, ROM in the MCPJ and IPJ according to normative paediatric data (Da Paz et al., 2016), CMCJ radiographs and stability testing, tip pinch and grip strength percentages to calculate the WIMEC and WIMMECSS scores.

All the children and caregivers completed together five Patient-Reported Outcomes Measurement Information System (PROMIS) domains validated from the age of 5 years: Parent Proxy Item Bank v2.0 Upper Extremity – Short Form 8a; Depressive Symptoms – Short Form 6a; Anxiety – Short Form 8a; Peer Relationships – Short Form 7a; and Parent Proxy Scale v1.0 – Global Health 7 + 2 (Cella et al., 2007). PROMIS domains are valid and recommended outcomes for children with congenital upper limb anomalies (CULA) (Wall et al., 2020). We converted raw scores to *t*-scores and compared the group medians to the reference (USA) populations from the PROMIS website.

The children aged >8 years (*n = *9) and all caregivers assessed the following statements or questions on a visual analogue scale (VAS; 0–100; 0 = sad face, 100 = happy face): the thumb works like a thumb; the thumb looks like a thumb; ‘How often do you (or does the child) use the thumb to pinch versus scissor pinch for small objects?’; ‘How often do you (or does the child) incorporate the thumb when holding larger objects like a bottle?’ (Zlotolow et al, 2014). Only the caregivers were asked ‘Has the child used the thumb more after surgery?’. The OT and surgeon also rated statements 1 and 2.

### Statistics

We report continuous outcomes as median and IQR, because the distribution of most outcomes was not normal. We performed stratified analyses according to severity of hypoplasia, a presumed prognostic factor (de Kraker et al., 2016; Vuillermin et al., 2016), but considered the sample sizes as being too small for formal statistical testing owing to the low power for detecting differences between the groups and the risk of false-positive (spurious) findings owing to chance.

## Results

All 20 children (6 girls and 14 boys) had a modified Bayne type N/0-1 RLD (James et al., 1999) in the 23 upper extremities that were included, and none were in need of ipsilateral wrist surgery (Table 2). In addition to the three children with bilateral FDS opponensplasties, nine had bilateral RLD, including two with a contralateral RLD Bayne type 4. Twelve children had other congenital anomalies, and six had a known syndrome, including two children with Fanconi anaemia (Supplementary Table S1, available online).

**Table 2.** Age at surgery and at follow-up and characteristics for radial longitudinal deficiency in the two thumb groups.

	Manske type II (9 thumbs in 7 children)	Manske type IIIa (14 thumbs in 13 children)
Age at operation (years)	3.7 (2.8 to 6.1)	3.3 (2.5 to 4.6)
Age at follow-up (years)	6.5 (5.7 to 8.9)	8.0 (5.9 to 12.0)
Follow-up time (years)	2.7 (1.7 to 3.5)	4.6 (2.5 to 9.7)
Ipsilateral Bayne classification modified by James et al. (1999) (*n*)		
N/0	6	7
I	3	7

Data are presented as median (IQR). Both thumbs in three children were included in the study. Two of these children had bilateral type II thumbs, and one child had bilateral type IIIa thumbs.

Two children had had secondary bilateral first web releases for recurring tightness before follow-up. We found no complications in the medical records or adverse events reported by the patients or their caregivers.

**Figure 3.**
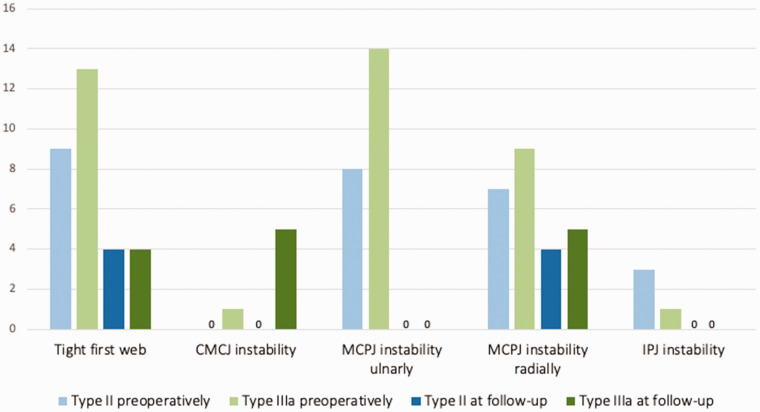
Assessment of the first web and the thumb joints before operation and at follow-up. CMCJ: carpometacarpal joint; IPJ: interphalangeal joint; MCPJ: metacarpophalangeal joint; RCL: radial collateral ligament; UCL: ulnar collateral ligament.

At follow-up, all the MCPJs were stable on the ulnar side, whereas nine were unstable radially (Figure 3; Table 3). None of the children with radial MCPJ instability or their caregivers reported a specific functional impairment related to this finding, and none needed additional stabilizing surgery. At follow-up, all four reconstructed IPJs were stable, but five type IIIa thumbs had a positive dorsal shift at follow-up assessment (Figure 3).

**Table 3.** Metacarpophalangeal joint stability preoperatively and at follow-up.

	Manske II (9 thumbs)	Manske IIIa (14 thumbs)
Preoperative description
Multidirectional instability	6	9
Ulnar instability only	2	5
Radial instability only	1	0
At follow-up
Ulnar instability	0	0
Radial instability	4	5
Radial collateral ligament reconstructed	4	3
Radial instability not preoperatively detected	0	2

The surgeon judged eight first webs to be tight (Figure 3), but only one child and his caregiver wanted to schedule surgery. T-GAP and WIMEC/WIMMECSS scores, thumb motion and strength are reported in Table 4.

**Table 4.** Functional and objective outcomes.

	Manske type II thumbs (*n = *9)	Manske type IIIa thumbs (*n = *14)
T-GAP score (0–63; 63=best)	45 (40 to 47)	45 (38 to 49)
WIMEC score (0–25; 25=best)	20 (20 to 21)	20 (18 to 21)
WIMMECSS score (0–40; 40=best)	30 (30 to 33)	30 (27 to 32)
Palmar abduction (degrees)	45 (37 to 49)	49 (41 to 61)
Palmar adduction (degrees)^a^	0 (0 to −18)	−8 (0 to −21)
Kapandji score (1–10; 10=best)	8 (7 to 8)	8 (7 to 9)
Radial abduction (degrees)	35 (29 to 40)	32 (24 to 46)
Retropulsion (mm)	3 (0 to 19)	2 (0 to 7)
MCPJ		
Flexion (degrees)	28 (5 to 41)	31 (8 to 46)
Extension (degrees)	6 (2 to 17)	2 (−7 to 23)
IPJ		
Flexion (degrees)	30 (13 to 42)	21 (12 to 31)
Extension (degrees)	−8 (−20 to 0)	0 (−14 to 3)
Grip strength (%)	57 (48 to 62)	52 (31 to 63)
Tip pinch (%)	46 (30 to 53)	36 (28 to 57)
Lateral pinch (%)	25 (19 to 33)	27 (17 to 42)
Palmar pinch (%)	38 (33 to 44)	34 (22 to 55)

Data are presented as median (IQR). All measurements of range of motion represent the children’s active abilities. Strength values are given as percentages of age- and sex-adjusted normative values.

a Palmar adduction of 0° represents full thumb adduction to the index.

IPJ, interphalangeal joint; MCPJ: metacarpophalangeal joint; T-GAP: Thumb Grasp and Pinch Assessment; WIMEC: an acronym for W = first web, I = intrinsic function, M = MCPJ stability, E = extrinsic function, C = CMCJ stability; WIMMECSS: an extended WIMEC score that includes measurements of range of motion (M_2_, mobility) and strength (S_1_, pinch; S_2_, grip).

We found a median PROMIS upper extremity *t*-score of 37 (IQR: 28 to 42) in type II thumbs and 35 (IQR: 32 to 50) in type IIIa thumbs, indicating a moderately reduced function. *t*-scores were normal for the other PROMIS domains (Supplementary Table S2, available online). The VAS assessments are presented in Supplementary Table S3 (available online).

## Discussion

We found satisfactory MCPJ stability after ligament reconstruction with FDS-ring tendon slips in both type II and IIIa hypoplastic thumbs, including those that were multidirectionally unstable before operation. The patient-reported and assessor-measured outcomes were also good after FDS opponensplasty.

The main strengths of our study were the consecutive cohort, preventing selection bias and a low attrition bias. As in earlier studies, the main limitations were the cohort size and the lack of preoperative outcome measures. We cannot rule out performance bias, as the assessors had treated most patients.

The successful stabilization of the ulnar side but with remaining radial instability in almost half of the multidirectionally unstable MCPJs might be because we routinely reconstructed the UCLs first and thus did not obtain correct tension in the RCLs. We have chosen this reconstruction sequence because the UCL is the most important MCPJ ligament for grip stability. Our proportion of stable joints after ligament reconstruction was comparable to the findings in two studies with an identical ligament insertion technique (Christen and Dautel, 2013; de Kraker et al., 2016), and one study with a slightly different tendon slip insertion technique with two bone tunnels (Smith et al., 2012). One study has reported little success with FDS tendon slips for ligament reconstruction in four patients (Mende et al., 2022). The largest FDS opponensplasty study did not report on MCPJ stability but concluded from the low number of reoperations that stability was adequate (Vuillermin et al., 2016). Others have suggested that MCP fusion or chondrodesis could be beneficial in multidirectionally unstable type IIIa thumbs (de Kraker et al., 2016; Smith et al., 2012; Tonkin, 2014). The different success rates in ligament stabilization might be related to different surgical techniques and to the definition of stability. The lack of a validated and reliability-tested definition of thumb joint stability has been discussed (Mende et al., 2019, 2022; Mende and Tonkin, 2021). Assessing thumb joint stability in small children is difficult, and not all authors have reported their methods. Some have defined instability with cut-off values of a yield of more than 20° (Smith et al., 2012) or 30° (de Kraker et al., 2016; Mende et al., 2022) in deviation stress testing measured with a goniometer. Engelhardt et al. (2016) tested 25 normal thumbs in children and found mean ulnar and radial MCPJ deviations of 25° and 30°, respectively, and a variability between 10° and 55°. We decided to use the surgeons’ subjective assessment owing to the lack of a valid cut-off value, high measurement variability among normal hands and the risk of low reliability in goniometer stress measurements in children. We believe this is how the indication for surgery is established in most hand units, and by mirroring daily clinical practice, we aimed to increase the generalizability of our findings.

A postoperative positive dorsal shift of the CMCJ has also been reported previously (de Kraker et al., 2016; Mende et al., 2022). In two alternatives to Manske and McCarroll’s (1992) modification of the Blauth classification, some authors have suggested a separate subtype (IIC) for thumbs with a clinically unstable and/or immobile CMCJ with or without radiological loss of the proximal flare of the first metacarpal base (Smith et al., 2012; Tonkin, 2014).

We found no detailed reports on the clinical assessment of the first web. There is no agreement on a clinical definition of a tight first web in children, and again we decided to use the surgeon’s subjective assessment. We did not note web tightness at follow-up as a surgical complication, as not all webs can be completely corrected with primary surgery, and recurrence is common in growing children as scar tissue is less flexible.

The T-GAP scores we report were predictably better than those reported after index finger pollicization (Kollitz et al., 2018). The ROM and strength outcomes were comparable to previous studies (Christen and Dautel, 2013; de Kraker et al., 2016; Mende et al., 2022; Vuillermin et al., 2016). The WIMEC/WIMMECSS instrument has been described only recently and awaits proper validation and reliability testing. The PROMIS outcomes were comparable to those reported for ADM opponensplasties (Wall et al., 2017). The outcomes of our questions assessed by VAS were comparable to reports from FDS (de Kraker et al., 2016) and ADM opponensplasty studies (Mende et al., 2022; Wall et al., 2017).

Our study indicates that FDS tendon slips are adequate to stabilize most MCPJs, but we cannot rule out an occasional need for primary or secondary joint fusion. The earlier treatment in our unit of mildly affected hypoplastic thumbs with instability and partial thenar aplasia was a ligament reconstruction with free tendon grafts. Palmar abduction and opposition are present in these thumbs, but strength is lacking. Based on the good outcomes in our study, we have expanded our indications of FDS opponensplasties to include these thumbs to strengthen them.

## Supplemental Material

sj-pdf-1-jhs-10.1177_17531934231187813 - Supplemental material for Satisfactory thumb metacarpophalangeal joint stability after ligament reconstruction with flexor digitorum superficialis in children with radial longitudinal deficiency Click here for additional data file. Supplemental material, sj-pdf-1-jhs-10.1177_17531934231187813 for Satisfactory thumb metacarpophalangeal joint stability after ligament reconstruction with flexor digitorum superficialis in children with radial longitudinal deficiency by Ida Neergård Sletten, Jarkko Jokihaara, Anne Birgit Stavenes and Mona Irene Winge in Journal of Hand Surgery (European Volume)

sj-pdf-2-jhs-10.1177_17531934231187813 - Supplemental material for Satisfactory thumb metacarpophalangeal joint stability after ligament reconstruction with flexor digitorum superficialis in children with radial longitudinal deficiency Click here for additional data file. Supplemental material, sj-pdf-2-jhs-10.1177_17531934231187813 for Satisfactory thumb metacarpophalangeal joint stability after ligament reconstruction with flexor digitorum superficialis in children with radial longitudinal deficiency by Ida Neergård Sletten, Jarkko Jokihaara, Anne Birgit Stavenes and Mona Irene Winge in Journal of Hand Surgery (European Volume)

sj-pdf-3-jhs-10.1177_17531934231187813 - Supplemental material for Satisfactory thumb metacarpophalangeal joint stability after ligament reconstruction with flexor digitorum superficialis in children with radial longitudinal deficiency Click here for additional data file. Supplemental material, sj-pdf-3-jhs-10.1177_17531934231187813 for Satisfactory thumb metacarpophalangeal joint stability after ligament reconstruction with flexor digitorum superficialis in children with radial longitudinal deficiency by Ida Neergård Sletten, Jarkko Jokihaara, Anne Birgit Stavenes and Mona Irene Winge in Journal of Hand Surgery (European Volume)
