# Supplementary material for: Satisfactory thumb metacarpophalangeal joint stability after ligament reconstruction with flexor digitorum superficialis in children with radial longitudinal deficiency
Source: J Hand Surg Eur Vol. 2023 Jul 13;48(11):1151–8. doi: 10.1177/17531934231187813 (PMC10668531; doi:10.1177/17531934231187813)
Supplement: sj-pdf-1-jhs-10.1177_17531934231187813 - Supplemental material for Satisfactory thumb metacarpophalangeal joint stability after ligament reconstruction with flexor digitorum superficialis in children with radial longitudinal deficiency [file sj-pdf-1-jhs-10.1177_17531934231187813.pdf]

Supplementary Table 1. Other congenital anomalies and syndromes in the 20 children.

	Manske type II (7 children)	Manske type IIIa (13 children)
Other congenital anomalies (n)		
Cardiac	2	4
Renal	2	2
Cleft palate		3
Pancytopenia	1	1
Other*	1	5
Syndromes (n)		
15q26q microdeletion syndrome	1	
Di Georges syndrome		1
Fanconi anemia	1	1
Nager syndrome		1
VACTERL association		1

*Café au lait skin spots, developmental delay, ear anomaly, sacral cyst, torticollis, vertebral anomalies

VACTERL: Vertebral defects, Anal atresia, Cardiac defects, Trachea-Esophageal fistula, Renal anomalies, and Limb abnormalities.
