# Supplementary material for: Satisfactory thumb metacarpophalangeal joint stability after ligament reconstruction with flexor digitorum superficialis in children with radial longitudinal deficiency
Source: J Hand Surg Eur Vol. 2023 Jul 13;48(11):1151–8. doi: 10.1177/17531934231187813 (PMC10668531; doi:10.1177/17531934231187813)
Supplement: sj-pdf-2-jhs-10.1177_17531934231187813 - Supplemental material for Satisfactory thumb metacarpophalangeal joint stability after ligament reconstruction with flexor digitorum superficialis in children with radial longitudinal deficiency [file sj-pdf-2-jhs-10.1177_17531934231187813.pdf]

Supplementary Table 2. Patient-Reported Outcomes Measurement Information System (PROMIS) parent proxy domains t-scores.

	Children with Manske type II thumbs (n=7)	Children with Manske type IIIa thumbs (n=13)
Upper extremity 8a v2.0	37 (28 to 42)	35 (32 to 50)
Depressive symptoms 6a v2.0	48 (45 to 57)	48 (39 to 57)
Anxiety 8a v2.0	49 (35 to 60)	45 (35 to 58)
Peer relationships 7a v2.0	49 (39 to 51)	51 (40 to 62)
Global health 7 v1.0	52 (44 to 55)	47 (40 to 57)

All continuous outcomes are reported as medians (interquartile ranges).

All domains have a mean score of 50 and a standard deviation of 10. Higher t-scores mean that an individual or a group has more of the measured concept than the reference (e.g., better upper extremity function or more depressive symptoms). Cut points for normal, mild, moderate, and severe are >45, >40, >30, and <30 for Upper extremity and <50, <55, <65, and >65 for Depressive Symptoms and Anxiety. Cut points for excellent, good, fair, and poor are >60, >40, >30, and <30 for Peer Relationships. Cut points for good, fair, and poor are >45, >36, and <36 for Global Health.
