# Supplementary material for: Satisfactory thumb metacarpophalangeal joint stability after ligament reconstruction with flexor digitorum superficialis in children with radial longitudinal deficiency
Source: J Hand Surg Eur Vol. 2023 Jul 13;48(11):1151–8. doi: 10.1177/17531934231187813 (PMC10668531; doi:10.1177/17531934231187813)
Supplement: sj-pdf-3-jhs-10.1177_17531934231187813 - Supplemental material for Satisfactory thumb metacarpophalangeal joint stability after ligament reconstruction with flexor digitorum superficialis in children with radial longitudinal deficiency [file sj-pdf-3-jhs-10.1177_17531934231187813.pdf]

Supplementary table 3. Visual analog scale assessments of statements/questions regarding thumb function and appearance.

	Manske type II thumbs (n=9)*	Manske type IIIa thumbs (n=14)*
Works like a thumb		
Patient	75	50 (17 to 87)
Caregiver	75 (52 to 86)	61 (26 to 79)
Surgeon	82 (76 to 95)	80 (69 to 89)
Occupational therapist	87 (49 to 99)	81 (63 to 95)
Looks like a thumb		
Patient	74	82 (41 to 94)
Caregiver	84 (68 to 91)	73 (38 to 80)
Surgeon	95 (83 to 99)	81 (63 to 92)
Occupational therapist	81 (69 to 94)	84 (68 to 93)
How often do you/does the child use the thumb to pinch versus scissor pinch for small objects?		
Patient	96	60 (5 to 98)
Caregiver	88 (70 to 97)	60 (27 to 88)
How often do you/does the child incorporate the thumb when holding larger objects like a bottle?		
Patient	84	60 (18 to 91)
Caregiver	90 (82 to 98)	92 (67 to 97)

Has the child used the thumb more after surgery?		
Caregiver	72 (64 to 92)	95 (56 to 99)

All continuous outcomes are reported as medians (interquartile ranges).

*The VAS questions were answered by 3 children >8 years with Manske type II and 6 children >8 years with Manske type IIIa thumbs, and the OT assessed all but 1 type II thumb. The caregivers and the surgeon assessed all thumbs.
